# Efficacy and Safety of Intra-articular Platelet-Rich Plasma (PRP) Versus Corticosteroid Injections in the Treatment of Knee Osteoarthritis: A Systematic Review of Randomized Clinical Trials

**DOI:** 10.7759/cureus.80948

**Published:** 2025-03-21

**Authors:** Diego Ivan Diaz Haaz, Oswaldo Rizo Castro

**Affiliations:** 1 Faculty of Human Medicine "Dr. Manuel Velasco Suárez" Campus II, Universidad Autónoma de Chiapas, Chiapas, MEX; 2 Department of Traumatology and Orthopaedics, Antiguo Hospital Civil de Guadalajara "Fray Antonio Alcalde", Guadalajara, MEX

**Keywords:** corticosteroid, intra-articular injection, knee osteoarthritis, oa, orthobiologic, osteoarthritis, platelet-rich plasma, prp

## Abstract

Osteoarthritis (OA) is a prevalent condition that significantly impacts the quality of life due to pain and associated disability. Platelet-rich plasma (PRP) injections have emerged as a promising alternative treatment, though their efficacy and safety remain debatable. This systematic review aims to evaluate the efficacy and safety of PRP injections in patients with knee OA by analyzing randomized clinical trials (RCTs). A comprehensive search was conducted across PubMed, Cochrane Central Register of Controlled Trials (CENTRAL), and the Virtual Health Library (VHL) for studies published from 2019 to 2024, following the Preferred Reporting Items for Systematic Reviews and Meta-Analyses (PRISMA) guidelines. Data extraction and methodological quality assessment were performed using the Cochrane Risk of Bias Tool (RoB 2). Of the 129 studies identified, six met the inclusion criteria. The studies varied in sample size (36-80 patients) and PRP preparation methods. The results suggest that intra-articular (IA) PRP and corticosteroid (CS) injections are safe and effective for treating knee OA, reducing pain and improving symptoms. Some studies indicate that PRP injections may offer prolonged benefits, although there is no consensus on whether one treatment is superior. No serious adverse effects were reported, and the side effects observed were mild, suggesting a favorable safety profile. In conclusion, PRP is a viable therapeutic alternative for managing knee OA, with sustained benefits compared to CS. However, further research is needed to standardize protocols and assess long-term effects.

## Introduction and background

Osteoarthritis (OA) is a chronic, progressive, degenerative disorder primarily affecting the synovial joints, especially the knees, hips, hands, and spine. It is characterized by the progressive loss of articular cartilage, changes in the subchondral bone, osteophyte formation, and varying degrees of inflammation [1]. The prevalence of knee OA is increasing due to population aging and rising obesity rates. Clinically, it presents with pain, joint deformity, and limited mobility, often leading to disability [2]. In nonsurgical treatment, intra-articular (IA) corticosteroids (CS) are used when symptoms worsen despite the use of non-steroidal anti-inflammatory drugs (NSAIDs) [3]. While various molecules are available for IA application, platelet-rich plasma (PRP) has gained popularity as a promising alternative due to its potential to improve symptoms in OA [1].

CS exert immunosuppressive and anti-inflammatory effects. They act on nuclear steroid receptors, modifying mRNA and protein synthesis, generating alterations in the functions of immune cells and the levels of proinflammatory enzymes and cytokines. They reduce the production of interleukin 1 (IL-1), leukotrienes, prostaglandins, and metalloproteinases. CS injections are used to manage both acute and chronic inflammations and are recommended as a temporary treatment for acute episodes of OA [4,5].

When PRP is injected, growth factors are released, including platelet-derived growth factor (PDGF), transforming growth factor beta (TFG-β), vascular endothelial growth factor (VEGF), and anti-inflammatory cytokines. These factors play key roles in processes like cell proliferation, migration, differentiation, angiogenesis, and extracellular matrix (ECM) production [6-8]. PRP also contains adhesive proteins such as fibrin, fibronectin, and vitronectin, which form a fibrin gel that acts as a scaffold, enhancing the healing process [9,10]. Additionally, the functional effects of PRP in treating OA are attributed to its ability to modulate the inflammatory response by reducing proinflammatory cytokines through the inhibition of IL-1 and nuclear factor kappa-B, as well as contributing to joint balance and homeostasis [11,12]. Some authors suggest that PRP should be considered as the first-line option for IA injections, although its use remains a matter of debate [13]. Therefore, this systematic review aims to evaluate and synthesize the available evidence to determine which therapy offers the best clinical outcomes in managing knee OA.

## Review

Methods

Search Strategy

This systematic review was conducted following the 2020 Preferred Reporting Items for Systematic Reviews and Meta-Analyses (PRISMA) guidelines. A systematic literature search was conducted for studies published between 2019 and 2024 across the following databases: PubMed, Cochrane Central Register of Controlled Trials (CENTRAL), and the Virtual Health Library (VHL). Relevant articles were identified using the terms "platelet-rich plasma", "PRP", "knee osteoarthritis", "corticosteroids", "CS", and "randomized clinical trial", in combination with the Boolean operators (AND, OR). Medical Subject Headings (MeSH) in PubMed were also used to find additional relevant studies.

Eligibility Criteria

Clinical studies evaluating IA PRP injections for knee OA were included if they met the following criteria: (1) only randomized clinical trials (RCTs) in English or Spanish; (2) patients ≥ 18 years old with a diagnosis of knee OA (unilateral or bilateral); (3) intervention group receiving IA PRP; (4) comparison group receiving IA CS injections; and (5) outcomes focusing on clinical efficacy and adverse effects. Exclusion criteria were as follows: (1) studies not related the treatment of knee OA; (2) animal studies; (3) studies where PRP and CS were not evaluated as primary treatments or where specific data could not be extracted for this comparison; (4) duplicate publications, secondary publications, or articles with similar data; (5) review articles, meeting abstracts, case reports, letters to the editor, or comments; and (6) articles accessible through the searched databases.

Outcome Assessment

We compared the results of the included studies using the most common outcome scales: the visual analog scale (VAS) and the Western Ontario and McMaster Universities Osteoarthritis Index (WOMAC).

Data Extraction

Data were extracted into a predefined spreadsheet, with the following study characteristics: (1-3) author, year, and title of the study; (4) number of patients randomized; (5) number of patients analyzed; (6) gender; (7) body mass index, (8) age; (9) grade of osteoarthritic lesion; and (10) follow-up period.

Methodological Quality Assessment

The methodological quality of the included studies was assessed using the Cochrane Risk of Bias Tool (RoB 2). This tool analyzes five main domains to identify potential biases: randomization and concealment, deviations from planned interventions (including blinding and protocol adherence), missing outcome data (completeness of follow-up), bias in outcome measurement (blinding of evaluators), and bias in outcome selection and reporting (selective reporting).

Results

Study Selection

**Figure 1:**
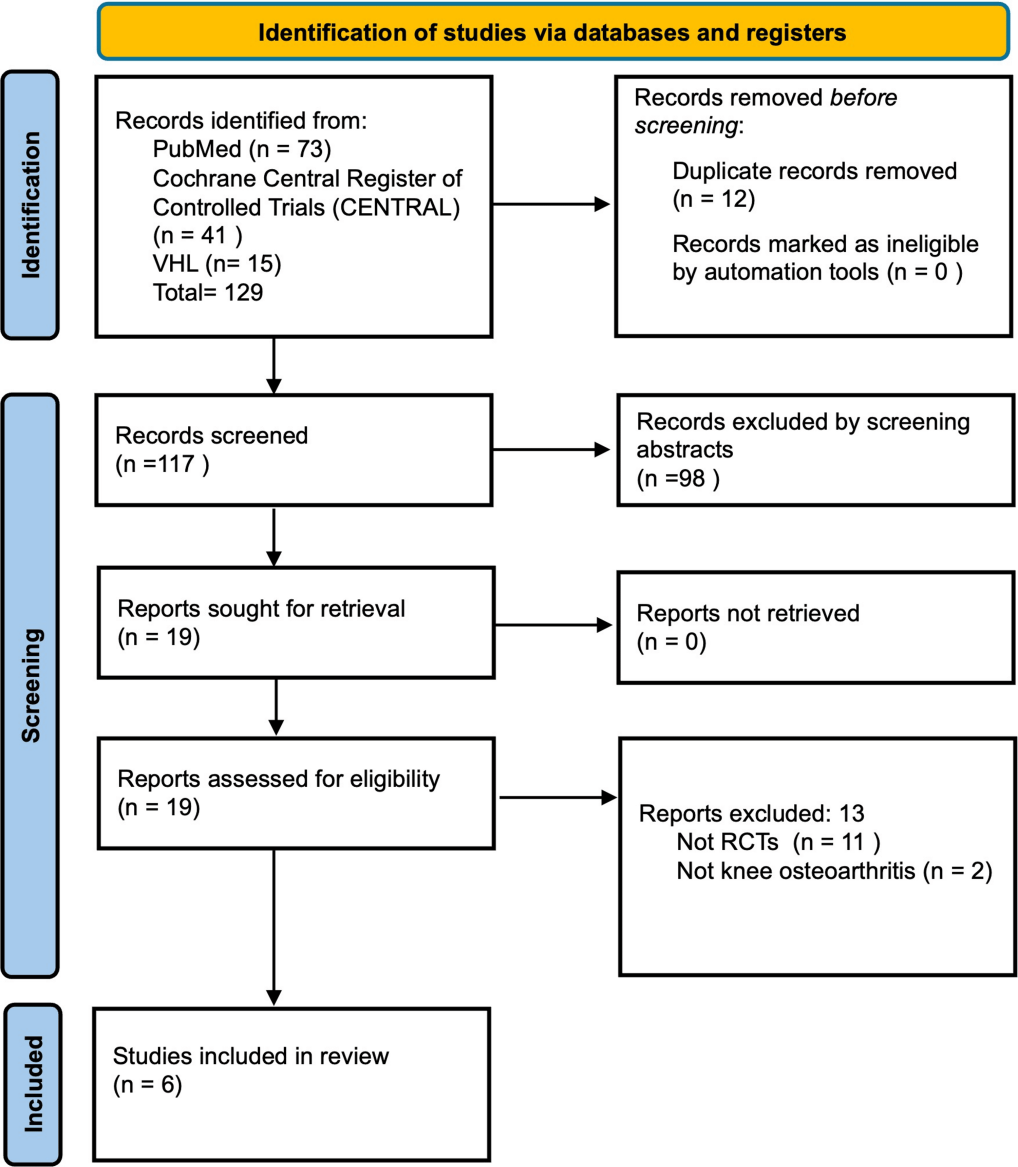
PRISMA flow diagram of the study selection process PRISMA: Preferred Reporting Items for Systematic Reviews and Meta-Analyses.

The search identified 129 studies (73 from PubMed, 41 from CENTRAL, and 15 from VHL). After removing 12 duplicates, 98 studies were excluded based on abstract review. Of the remaining 19 studies, 13 were excluded after full-text review, leaving six studies for inclusion [14-19]. Therefore, our systematic review synthesized data from six clinical trials comparing the efficacy and safety of IA PRP and CS for the treatment of knee OA. The process is outlined in the PRISMA flow diagram in Figure 1. The characteristics of the studies and patients are summarized in Table 1.

**Table 1:** Study characteristics CS: corticosteroids, PRP: platelet-rich plasma, M: male, F: female, BMI: body mass index, SD: standard d, K-L: Kellgren-Lawrence.

Author, year	Follow-up period (months)	Analyzed, N PRP	Analyzed, N CS	Gender, M:F, n PRP	Gender, M:F, n CS	Age, years, mean, N PRP	Age, years, mean, N CS	BMI, mean/SD, N (%) PRP		BMI, mean/SD, N (%) CS	K-L, degrees, n PRP	K-L, degrees, n CS
Elksniņš-Finogejevs et al. [14]	12	19	17	17:3	15:5	66.4 ± 8.4	70.2 ± 9.2	28.6 ± 5.0		30.5 ± 5.8	II: 5, III: 15	II: 6, III: 14
Pretorius et al. [15]	6	29	29	14:17	14:17	63.8 ± 9.7	63.8 ± 9.7	32.7 ± 4.9		32.7 ± 4.9	II: 8, III: 21	II: 10, III: 19
Nunes-Tamashiro et al. [16]	12	34	33	30:4	30:3	67.6 ± 7.4	65.8 ± 6.1	29.2 ± 3.2		29.5 ± 4.5	II: 14, III: 20	II: 16, III: 17
de Menezes Freire et al. [17]	6	25	25	42:8	42:8	64.15 ± 8.02	60.21 ± 5.92	19 obese, 6 not	22 obese, 3 not		I: 0, II: 10, III: 11, IV: 4	I: 1, II: 10, III: 14, IV: 0
Huang et al. [18]	12	40	40	25:15	21:19	54.5 ± 1.2	54.3 ± 1.4	25.23 ± 4.15		24.56 ± 3.62	I/II	I/II
Tschopp et al. [19]	24	30	30	17:13	14:16	62.0 ± 8.9	59.0 ± 11.9	26.0 ± 5.5		27.0 ± 4.7	I: 9, II: 5, III: 7	I: 4, II: 6, III: 15

Efficacy

PRP and pain: Two studies showed that PRP injections reduced pain in most patients up to six months [15,17], while two additional studies mentioned an improvement in pain up to 12 months [14,18]. Tschopp et al. [19] reported a slight improvement in the PRP group up to 24 months.

PRP and function: Functional improvement was consistently assessed using the WOMAC scale across these clinical trials [15-19]. Most studies reported functional improvement in patients receiving PRP injections [16-18], though Tschopp et al. [19] found no significant functional improvement.

CS: CS injections were also effective in reducing pain and improving function, but only in the short term [14-19]. However, PRP outperformed CS in long-term pain reduction [14,15,17,18]. 

Safety

Both PRP and CS demonstrated satisfactory safety profiles, with no serious adverse events reported in any of the studies analyzed. Minor side effects, such as pain, nausea, or localized discomfort, were transient and resolved without further treatment [14-19]. 

Methodological Quality and Risk of Bias

**Figure 2:**
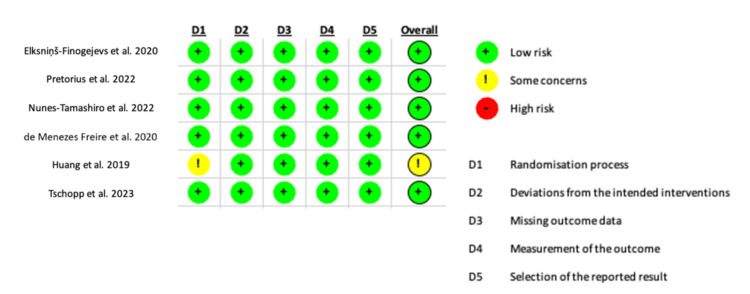
Risk of bias (RoB 2) assessment results

We utilized the Cochrane Risk of Bias Tool (RoB 2) to assess potential biases, identifying issues such as insufficient information on randomization and blinding in some cases, as well as variations in the duration of follow-up. The studies by Elksniņš-Finogejevs et al. [14], Pretorius et al. [15], Nunes-Tamashiro et al. [16], de Menezes Freire et al. [17], and Tschopp et al. [19] were methodologically sound with generally low risk of bias. In contrast, Huang et al. [18] showed limitations, such as unclear blinding. However, all included studies yielded consistent and robust results. Figure 2 summarizes our assessment of the analyzed studies.

Discussion

This systematic review evaluates the efficacy and safety of PRP versus injected CS for the treatment of knee OA. Analysis of the six included studies provides relevant findings on the effects of both treatments in terms of efficacy and safety. 

Efficacy Comparison

Elksniņš-Finogejevs et al. [14]: This study demonstrated that both groups were effective in reducing pain and improving knee function after the first week. The difference was significant between groups (p = 0.0002). The greatest improvement in VAS occurred at three months in the PRP group (mean - 4.6 ± 1.6; - 77%) and at one month in the CS group (- 3.4 ± 1.2; - 58%). However, the PRP group showed a sustained improvement in pain up to seven months, while the CS group worsened after 15 weeks of treatment. Improvement of knee function was observed in both groups up to 5-15 weeks, with no significant difference between groups (p > 0.05).

Pretorius et al. [15]: This study found a statistically significant improvement in pain scores over the 6-month follow-up for both treatment groups (p = 0.005). Regarding improvement in function, the data showed no evidence of a significant difference in treatment effects (PRP vs. CS) at the different time points assessed for the total WOMAC score (p = 0.84).

Nunes-Tamashiro et al. [16]: This randomized clinical trial found no statistically significant difference between the two groups in terms of pain reduction over 12 months (p = 0.433). The PRP group showed better function according to WOMAC at 12 months, with a statistically significant difference (p < 0.001).

de Menezes Freire et al. [17]: This study reported that there were statistically significant differences in favor of the PRP therapy for pain reduction at six months (p < 0.001). Functional improvements measured by WOMAC were more significant at six months for the PRP group (p < 0.001).

Huang et al. [18]: In this study, both groups obtained benefits in pain reduction up to 12 months, which was significant (p < 0.01), compared to pretreatment. Functional improvements, measured by WOMAC, showed a significant difference in favor of the PRP group up to 12 months (p < 0.01).

Tschopp et al. [19]: This study found no significant difference in pain reduction between the two groups (p = 0.75). In the WOMAC, with respect to function, neither group showed a significant difference, and there was no evidence that drug effects differed over time (p = 0.31).

In summary, Elksniņš-Finogejevs et al. [14] demonstrated that both groups were effective in reducing pain and improving function from the first week. Pretorius et al. [15] found that there were statistical improvements for both groups in pain score; in terms of function, the data did not show a statistically significant difference. Nunes-Tamashiro et al. [16] found no significant difference in both groups in pain reduction; however, in the PRP group, an improvement in function was observed at 52 weeks. de Menezes Freire et al. [17] demonstrated significant differences in favor of PRP for both pain and function. Huang et al. [18] reported favorable results; however, in terms of function, PRP obtained better results. Tschopp et al. [19] found no difference between both treatment groups. Table 2 shows the comparison of efficacy between the studies.

**Table 2:** Comparison of efficacy between studies WOMAC: Western Ontario and McMaster Universities Osteoarthritis Index.

Study	Pain reduction	Functional improvement
Elksniņš-Finogejevs et al. [14]	Significant at 12 months (p = 0.0002)	Not significant between groups (p > 0.05)
Pretorius et al. [15]	Significant at six months (p = 0.005)	Not significant between groups (WOMAC, p = 0.84)
Nunes-Tamashiro et al. [16]	Not significant at 12 months (p = 0.433)	Significant at 12 months (WOMAC, p < 0.001)
de Menezes Freire et al. [17]	Significant at six months (p < 0.001)	Significant at six months (WOMAC, p < 0.001)
Huang et al. [18]	Significant at 12 months (p < 0.01)	Significant at 12 months (WOMAC, p < 0.01)
Tschopp et al. [19]	Not significant in both groups (p = 0.75)	Not significant (WOMAC, p = 0.31)

Safety Comparison

Elksniņš-Finogejevs et al. [14]: This study included 36 patients. Fifteen patients in the PRP group recorded mild synovitis in the first week that resolved spontaneously. 

Pretorius et al. [15]: In this study involving 58 patients, none presented side effects after receiving the injections.

Nunes-Tamashiro et al. [16]: With a total of 67 participants, the study did not report any serious adverse events related to PRP and CS injections, further reinforcing the safety profile in both groups.

Freire et al. [17]: This study involved 50 patients. No adverse effects occurred in any group, suggesting a robust safety profile for PRP injections.

Huang et al. [18]: In this study, which involved 80 patients, minor side effects, such as pain, nausea, and dizziness, were observed in three patients in the CS group and five in the PRP group.

Tschopp et al. [19]: With 60 patients, this study reported three secondary events in the CS group: one patient had facial flushing, palpitations, and knee joint swelling at three months and the other experienced nausea and vomiting immediately after applying CS. Table 3 shows a summary of the safety comparison in the included studies.

**Table 3:** Comparison of safety between studies PRP: platelet-rich plasma, CS: corticosteroids.

Study	Sample size	Adverse events
Elksniņš-Finogejevs et al. [14]	36	Fifteen patients in the PRP group had mild synovitis
Pretorius et al. [15]	58	No complications reported
Nunes-Tamashiro et al. [16]	67	No complications reported
de Menezes Freire et al. [17]	50	No complications reported
Huang et al. [18]	80	Minor side effects (pain, nausea, and dizziness): two patients in the CS group and five in the PRP group
Tschopp et al. [19]	60	One patient in the CS group presented facial redness, palpitations, and joint swelling, and the other presented with nausea and vomiting

Limitations and variability

Study Design Variability

The included studies showed remarkable heterogeneity in terms of PRP preparation methods and duration of follow-up. This variability makes direct comparisons of results difficult and highlights the need for standardized protocols. Factors such as PRP concentration, preparation, and processing techniques may influence treatment efficacy and safety. In addition, differences in the characteristics of the populations studied and methodological variations limit the generalizability of the findings.

Future Research Directions

Standardization of protocols: It is essential that future research focus on standardizing PRP preparation and administration procedures to facilitate more accurate comparisons between studies.

Personalized treatment strategies: Determining individual factors such as age, degree of disease progression, and the presence of comorbidities could help optimize the selection of the most appropriate treatment between PRP and CS.

## Conclusions

The comparative analysis of the clinical trials supports the use of PRP as an effective and safe therapeutic alternative for managing knee OA. While CS injections provide immediate symptomatic relief, PRP has demonstrated sustained benefits in pain reduction and long-term functional improvement, with no serious adverse effects. However, the variability in PRP preparation and administration protocols highlights the need for standardization to optimize its effectiveness and allow for more accurate comparisons in future studies. Furthermore, it is essential to continue investigating its long-term effects and its potential application in different patient subgroups to develop personalized treatment strategies.

## References

[1] Testa G, Giardina SM, Culmone A, Vescio A, Turchetta M, Cannavò S, Pavone V (2021). Intra-articular injections in knee osteoarthritis: a review of literature. J Funct Morphol Kinesiol.

[2] Geng R, Li J, Yu C (2023). Knee osteoarthritis: current status and research progress in treatment (review). Exp Ther Med.

[3] Hussain SM, Neilly DW, Baliga S, Patil S, Meek R (2016). Knee osteoarthritis: a review of management options. Scott Med J.

[4] Richard MJ, Driban JB, McAlindon TE (2023). Pharmaceutical treatment of osteoarthritis. Osteoarthritis Cartilage.

[5] Mora JC, Przkora R, Cruz-Almeida Y (2018). Knee osteoarthritis: pathophysiology and current treatment modalities. J Pain Res.

[6] Rodríguez-Merchán EC (2022). Intra-articular platelet-rich plasma injections in knee osteoarthritis: a review of their current molecular mechanisms of action and their degree of efficacy. Int J Mol Sci.

[7] Wasserman A, Matthewson G, MacDonald P (2018). Platelet-rich plasma and the knee-applications in orthopedic surgery. Curr Rev Musculoskelet Med.

[8] Nguyen C, Lefèvre-Colau MM, Poiraudeau S, Rannou F (2016). Evidence and recommendations for use of intra-articular injections for knee osteoarthritis. Ann Phys Rehabil Med.

[9] Shahbaz A, Alzarooni A, Veeranagari VR (2024). Efficacy of platelet-rich plasma intra-articular injections in hip and knee osteoarthritis. Cureus.

[10] Sánchez M, Delgado D, Sánchez P (2014). Platelet rich plasma and knee surgery. Biomed Res Int.

[11] Szwedowski D, Szczepanek J, Paczesny Ł, Zabrzyński J, Gagat M, Mobasheri A, Jeka S (2021). The effect of platelet-rich plasma on the intra-articular microenvironment in knee osteoarthritis. Int J Mol Sci.

[12] Cai Z, Cui Y, Wang J (2022). A narrative review of the progress in the treatment of knee osteoarthritis. Ann Transl Med.

[13] Cook CS, Smith PA (2018). Clinical update: why PRP should be your first choice for injection therapy in treating osteoarthritis of the knee. Curr Rev Musculoskelet Med.

[14] Elksniņš-Finogejevs A, Vidal L, Peredistijs A (2020). Intra-articular platelet-rich plasma vs corticosteroids in the treatment of moderate knee osteoarthritis: a single-center prospective randomized controlled study with a 1-year follow up. J Orthop Surg Res.

[15] Pretorius J, Nemat N, Alsayed A, Mustafa A, Hammad Y, Shaju T, Nadeem S (2022). Double-blind randomized controlled trial comparing platelet-rich plasma with intra-articular corticosteroid injections in patients with bilateral knee osteoarthritis. Cureus.

[16] Nunes-Tamashiro JC, Natour J, Ramuth FM, Toffolo SR, Mendes JG, Rosenfeld A, Furtado RN (2022). Intra-articular injection with platelet-rich plasma compared to triamcinolone hexacetonide or saline solution in knee osteoarthritis: a double blinded randomized controlled trial with one year follow-up. Clin Rehabil.

[17] de Menezes Freire MR, da Silva PM, Azevedo AR, Silva DS, da Silva RB, Cardoso JC (2020). Comparative effect between infiltration of platelet-rich plasma and the use of corticosteroids in the treatment of knee osteoarthritis: a prospective and randomized clinical trial. Rev Bras Ortop (Sao Paulo).

[18] Huang Y, Liu X, Xu X, Liu J (2019). Intra-articular injections of platelet-rich plasma, hyaluronic acid or corticosteroids for knee osteoarthritis. A prospective randomized controlled study. Orthopade.

[19] Tschopp M, Pfirrmann CW, Fucentese SF (2023). A randomized trial of intra-articular injection therapy for knee osteoarthritis. Invest Radiol.

